# Proportion of early treatment seeking for sexually transmitted infections and associated factors among patients attending youth friendly service in Bahir Dar City health centers, Northwest, Ethiopia

**DOI:** 10.1186/s12978-024-01794-1

**Published:** 2024-06-15

**Authors:** Getachew Zeleke, Alemtshay Mekonnen, Tigist Abuhay, Muluken Chanie Agimas

**Affiliations:** 1 Han health center, Bahir Dar health department, Bahir Dar, Amhara region, Ethiopia; 2 Department of reproductive health, college of medicine and health sciences, Bahir Dar university, Bahir Dar, Bahir Dar, Ethiopia; 3 Department of Epidemiology and Biostatistics, institute of public health, college of medicine and health sciences, university of Gondar, Gondar, Ethiopia

**Keywords:** Early treatment, STI, Youths, Ethiopia

## Abstract

**Background:**

Sexually transmitted infection is a common public health issue of youths and is characteristically transmitted through sexual intercourse. Even though early treatment for sexually transmitted infection is very important to reduce further complications and economic burden, studies to identify the proportion and the possible factor of early treatment seeking is rare in Ethiopia.

**Objective:**

To assess the proportion of early treatment seeking for sexually transmitted infections and associated factors among patients attending youth friendly service in Bahir Dar city health centers, northwest, Ethiopia 2023.

**Method:**

Institutional based Cross-sectional study was used among 407 participants from April 25 to May 24 /2023. A systematic random sampling technique was used to select the participants. An interview-administered questionnaire was used for data collection, whereas Epi-data version 4.6.0.2 and the statistical package for statistical science version 23 were used for data entry and analysis respectively. A frequency table and bar chart were used for descriptive analysis. Multiple binary logistic regression was employed to identify the factors at *p*-value of <0.05. The necessary assumption of the model was also checked by the Hosmer and Lemishow test.

**Results:**

The response rate of this study was 391 (96.1%) and the proportion of early treatment for sexually transmitted infection was 108 (27.6%, 95%CI; 23-32). Good knowledge about sexually transmitted infection (AOR=1.98, 95CI%; 1.13-3.47) know about their HIV status (AOR=1.95, 95%CI; 1.13-3.36), perceive severity of sexually transmitted infection (AOR=11.23, 95%CI; 6.15-20.45), and not fear the stigma about being infected with sexually transmitted infection (AOR=2.29, 95%CI; 1.32-3.96) were the significantly associated factors for early treatment of sexually transmitted infection.

**Conclusion and recommendation:**

The proportion of early treatment for sexually transmitted infection in Bahir Dar city was low. Knowledge about STIs, testing/ knowing HIV status, perception of the severity about sexually transmitted infection, and fear of stigma about sexually transmitted infection were the statistically significant factors for early treatment of sexually transmitted infection. So the government better give attention to health education and other health promotion activities to increase the knowledge of youths about sexually transmitted infection and to change their perception of sexually transmitted infection.

## Introduction

Sexually transmitted infection is a common public health issues of youths and is characteristically transmitted through sexual intercourse that can either end or advanced into sexually transmitted disease [1]. Bacteria, viruses and parasites are responsible for STIs such as for gonorrhea, chlamydial infection, syphilis, trichomoniasis, chancroid, genital herpes, human immunodeficiency virus infection and hepatitis B infection [2].

Especially, STI affects the young and adolescents groups [3, 4]. Unless STIs patients seek treatment early, it is endanger to transmit the infection and can be increase the risk of complications [5]. Thus the transmission rate of the human immunodeficiency virus can be increased by because of the treatment of STIs is not early [6]. Unmanaged STIs could be causes of obstetrics and gynecologic complication, severe psychological problems and cervical cancer [7]. Additionally, not early treatment of these infection could lead to health and economic burden. Particularly, in less developed countries the impact of untreated STI accounts about 17% of all economic losses [8]. The World Health Organization (WHO) report showed that, about 374 million new cases of curable STI (syphilis, gonorrhea and chlamydia) has occurred in 2021 [9]. From this figure, 40% of the global burden of STI is from sub-Saharan Africa [10]. As report in Benishangul Ethiopia revealed, the proportion of early treatment for STI is 40.1% [11]. As evidences showed, patients with one or more sign/symptom of STI usually visit health institutions too late. Because of some STI symptoms causes embracement and so patients are favor to be have self-medication and access the drugs from drugs stores with very minimum counseling that leads to drug resistance [12]. The other reason for not treating STIs early is because social stigma [13].

Studies showed that early treatment seeking behavior of the STI can be influenced by several factors such as educational status [14–16], knowledge about STIs [11, 15, 17, 18], perception about STIs [19, 20]., fear of stigma [11, 14, 20], numbers of sexual partners [11]. The WHO set a strategy on STIs in 2016 to end up the epidemic of STIs between 2016- 2021 [21]. But treatment seeking for STIs is still too low. Even though there are key strategic objectives about the quality services of adolescent health in Ethiopia [22], there is no adequate studies about early treatment seeking of STIs to design evidence based prevention mechanisms, strategies and policy to promote early treatment seeking behavior of STIs. Therefore this study was aimed to assess the prevalence of early treatment seeking and its associated factors among youths in Bahir Dar health centers.

## Methods and materials

### Study design, setting and study period

Institutional based cross-sectional study was employed from April 25/2023 to May 24/2023 in Bahir Dar city. Bahir Dar city is located about 565 km to the Addis Ababa, a capital city of Ethiopia. Bahir Dar city is located in the North Western part of Ethiopia. In the city, 15-24 age group accounts about 78,930 [23]. In Bahir Dar city administration, there are 10 health centers namely Shumabo, Shimbit, Bahir Dar, Meshentie, Abay, Dagmawi minilik, Zegie, Zenzelima, Tis Abay and Han. All these institutions provides the youth friendly service in a separate room.

### Population

All patients with STI in youth friendly service of Bahir Dar city health centers were the source and study population. All patients of youth friendly service diagnosed with STI in Bahir Dar city health centers were included in the study.

### Variables

#### Dependent variable

Early treatment seeking for STI (Yes, No)

#### Independent variables

Socio-demographic variables**:** age, sex, ethnicity, religion, residence, educational status and wealth index.

Knowledge and perception about STI**:** knowledge about STI, perception towards STI (severity, treatment), fear of stigma and believe about STI treatment.

Behavioral factors: numbers of sexual partner, age at the first sex, being tested for HIV, media exposure and distance from the health facility.

#### Operational definitions

Time of health care seeking for STIs: The extent of time of health care seeking is define to the patient “How long day did you wait or postpone before seeking treatment at the health facility after noticing the first symptoms of sexual transmitted infection?” It has two response categories: Early health care seeking refers to patients who seek care and/or advice within 7 days of the onset of the STI symptoms. And Delayed/not early health care seeking refers to patients who seek care and/or advice after 7 days of the onset of the STI symptoms [11].

Multiple sexual partners**:** are defined as the behavior of a person with two or more sexual partners [24].

Knowledge about STIs**:** A mean score was used to determine the knowledge status of respondents on STIs. Respondents who score above the mean were categorized as having good knowledge and those who score equal to or below mean were categorized as having poor knowledge [14].

Patients with STIs**:** in this study referred as patients who presented with one or more of STI symptoms like urethral discharges, vaginal discharges, lower abdominal pain, penile ulcers or ulcers of the vulva or vagina, perineal ulcers, genital or perineal warts or painful micturition and other STI symptoms [25].

#### Sample Size determination

The required sample size was determined by considering the single population proportion of early treatment seeking 40.1% which was conducted in Benishangul Gumuz Ethiopia [14] with the confidence level (CL) of 95% and a margin of error 5% . Based on these information, the sample size was calculated as follows.

$$\begin{array}{c}{\text{n}}=\frac{(\mathrm{Z a}/2{)}^{2}\mathrm{ P }(1 -\mathrm{ P})}{{{\text{d}}}^{2}}\\ =\boldsymbol{ }\frac{(1.96{)}^{2}(0.401)(1-0.401)}{(0.05{)}^{2}}\\ \begin{array}{c}{\text{n}}=\boldsymbol{ }370\\ \mathrm{None response}= 370*10\mathrm{\%}=37\dots ..10\mathrm{\% non}-\mathrm{response rate}\\ \begin{array}{c}\mathrm{Final sample }= 370 +37\\ =407\end{array}\end{array}\end{array}$$

#### Sampling procedure and sampling technique

**Fig. 1:**
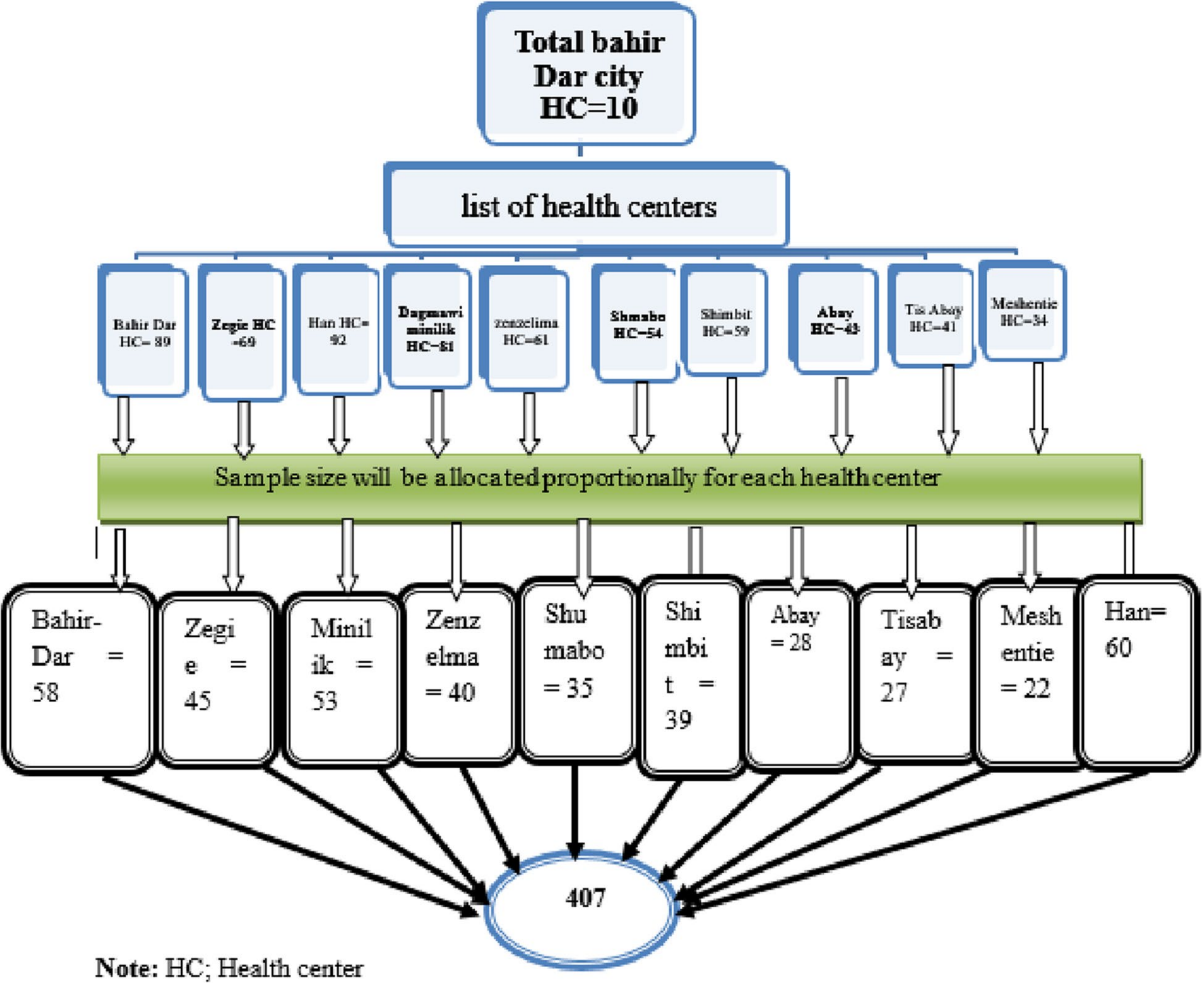
Schematic presentation of sampling technique and sampling procedure of patients/clients of youth friendly service in Bahir Dar city, 2023

First all health centers were identified namely Bahir Dar, Han, Shumabo, Meshentie, Tis Abay, Zenzelima, Dagmawi Minilik, Zegie, Shimbit and Abay and then all health centers were included in the study to be more representative and get adequate sample size. From each heath centers, numbers of participants was allocated proportionally to the size by using previous two consecutive months STI report of each health centers. Then to allocate the participants; “N” which was the number of patients treated in the previous two consecutive months and “n” was the number of samples allocated for a particular health centers then the interval (k) was determined by “N/n” and the random start was selected by lottery method (Fig. 1). Finally, a systematic random sampling method was used to select the eligible participants.

#### Data collection procedures and data quality assurance

A structured interview administered questionnaire was used to assess the proportion of early treatment seeking for STI and associated factors. Based on the objective of the study, the questionnaire was adapted from different litratures [11, 17, 19, 26]. The total duration of data collection was one month (from April 25/2023 to May 24/2023). Data were collected at the health facilities when they were coming to get a service. The social desirability bias was reduced by informing the purpose of the study carefully, making the questionnaire anonymous and collect the data by the health care providors who diagnosed them (to get the valid response). The questionnaire was first prepared in English language and then translated to the Amharic language, which was the local language of the study subjects and back to English by language experts to check their consistency and conceptual equivalence. Amharic version of the questionnaire was used to obtain data from participants. Ten nurses were recrieted for data collectors and monitored by two senior nurse supervisors. Each completed questionnaire was cheeked for coherence, completeness, consistency at the same time. The daily evaluation was performed to correct the problem that was face during the course of data collection and the pretest was conducted at Hamusit health center among 21 (5%) participants. One day intensive training was also given for both data collectors and supervisors.

#### Data processing and analysis

After cleaning and checking, data were coded and entered into Epi-data version 4.6.0.2 statistical software and exported to SPSS Version 23 statistical software for analysis. Descriptive statistics was summarized by using frequency, table and bar graph. All variables with a p-value of < 0.25 in the simple binary logistic regression analysis was kept for multivariable binary logistic regression analysis. A variable with a p-values of < 0.05 was considered as a statistically significant. Adjusted odds ratios with 95% CL was used to report the association between dependent and independent variables. The model fitness of binary logistic regression was checked by using Hosmer–Lemishow goodness of fit test with a p-value of > 0.05.

## Results

### Socio demographic characteristics of the participants

The response rate of this study was 391 (96.1%). Out of these, 249 (63.7%) of the respondents were female. Two hundred forty-two (61.9%) of the respondents were from urban residence. Furthermore, more than half, 272 (69.6%) of the respondents were orthodox religious followers (Table 1).

**Table 1:** Socio-demographic characteristics of youths in Bahir Dar city health centers, Northwest Ethiopia, 2023

**Variables**	**Category**	**Frequency**	**Percent (%)**
Sex	Male	142	36.3%
	Female	249	63.7%
Age	10-19 years	162	41.1%
	20-24 years	229	58.6%
	Urban	242	61.9%
Residence	Rural	149	38.1%
	No formal education	124	31.7%
Education status	Primary school	78	19.9%
	Secondary school	105	26.9%
	College and above	84	21.5%
Religion	Orthodox	272	69.6%
	Muslim	62	15.9%
	Protestant	44	11.3%
	Others^a^	13	3.3 %
Ethnicity	Amhara	329	84.1%
	Oromo	39	10%
Family wealth index	Tigre	4	1%
	Others^b^	18	4.9%
	Poor	64	33.3%
	Middle	45	23.4%
	Rich	83	43.3%

^a^Other: Catholic, Adventist

^b^Other: Kimant, gurage

### Knowledge and perception about STI

One hundred eighty-six (47.6%) of the participants were perceived STIs as a curable disease and about 207 (52.9%) of the participants had good knowledge about STIs. Furthermore, 217 (55.5%) of the participants had ever heard about STIs (Table 2).

**Table 2:** Knowledge and perception about STIs among youths in Bahir Dar city health centers, Northwest Ethiopia, 2023

**Variables**	**Category**	**Frequency**	**Percent (%)**
Perceiving STI curable	Yes	186	47.6%
What is the treatment	No	205	52.4%
	Traditional	115	61.8%
	Self-limited	91	48.9%
	Modern medicine	117	62.9%
	Holy water	113	60.8%
	Other^a^	91	48.9%
Knowledge about STI	Good	207	52.9%
	Poor	184	47.1%
Ever hear about STI	Yes	217	55.5%
	No	174	44.5%
Source of information about STI	Friend	104	48%
	Health worker	80	36.8%
	Spouse	20	9.2%
	Other specify	13	6%
Is STI preventable	Yes	278	71.1%
	No	113	28.9%
Prevented by	Abstain	181	65.1 %
	Condom	181	65.3%
	Avoid multiple sexuality	163	58.6%
Transmitted by	Blood transfusion	169	60.8%
	Cloth sharing	123	44.2%
	Mosquito bite	115	41.4%
	Kissing	117	42.1%
Sign symptom of STI	Loss of appetite	218	55.8%
	Discharge from genital area	232	59.3%
	Genital itching	230	58.8%
	Unable/painful urination	232	59.3%
	Loss of weight	213	54.5%
	Weakness	206	52.7%
	Pain during sex	221	56.5%
	Open sore/ulcer	206	52.7%
	Don’t know	83	21.2%
Know about HIV status	Yes	169	43.2%
Complication of STI	No	222	56.8%
	Infertility	231	59.1%
	Still birth	226	57.8%
	Ectopic pregnancy	211	54%
	Miscarriage	199	50.9%
	Cervical cancer	219	56%
Perception-about severity of STI			
	Don’t know	57	14.6%
	Not serious	223	57%
	Neutral	60	15.3%
	Very serious	108	27.7%
	Yes	236	60.4%
Fear of stigma about STI			
	No	155	39.6%

Other^a^= pray

### Sexual behavior and health service accessibility

About 209 (53.5%) of the participants were reported that distance as a big problem to access STI treatment. Two hundred (51.2%) of the participants were not used condom and 69 (34.5%) of them were because of the cost of condom. Furthermore, 251 (64.2%) of the respondents have had one sexual partner in the last 12 months (Table 3).

**Table 3:** Sexual behavior, media and health service accessibility among youths in Bahir Dar city health centers, Northwest, Ethiopia, 2023

**Variables**	**Category**	**Frequency**	**Percent (%)**
Attend-STI related message	Yes	135	34.5%
	No	256	65.5%
Distance is a big problem for STI treatment	Yes	209	53.5%
	No	182	46.5%
Ever use of condom	Yes	191	48.8%
	No	200	51.2%
	Restricted by my religion	71	35.5%
	Don’t know how to use	67	33.5%
Why not use condom?	Cost of service	69	34.5%
	My partner refuse	62	31%
	Reduce sensation	64	32%
	Ashamed to use	63	31.5%
	Not available during sex	71	35.5%
	Yes	265	67.8%
Sex-while symptomatic	No	126	32.2%
Sexual partner in the last 12 month	1	251	64.2%
	>=2	140	35.8%
Age of first sexual initiation	10-19 years	278	71.1%
	20-24years	113	28.9%

### Proportion of early treatment for STI

**Fig. 2:**
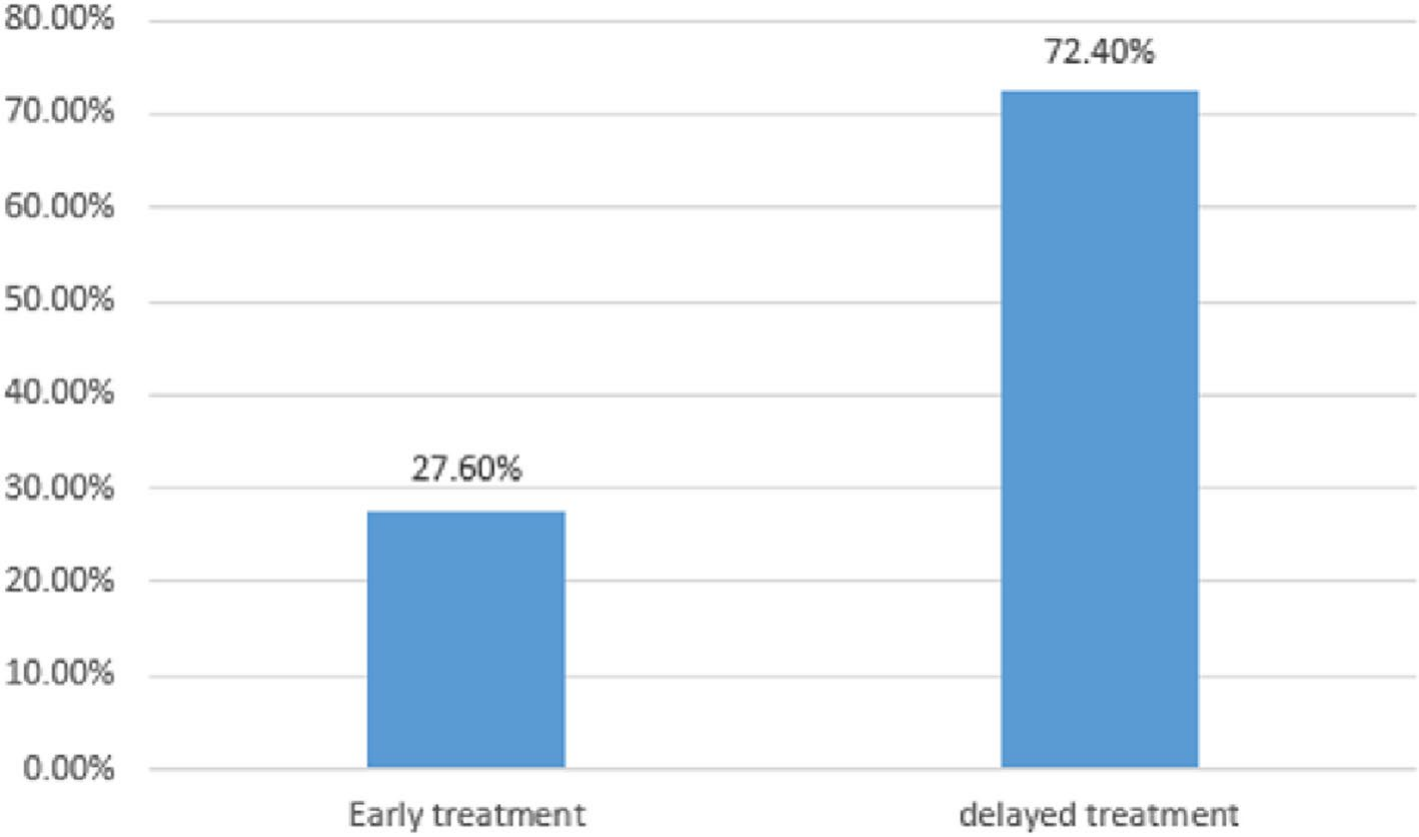
Proportion of early treatment for STI among youths in Bahir Dar city health centers, Northwest Ethiopia, 2023

In general, 27.6% (95%CI; 23-32) of the participants were found to be treated early for STI (Fig. 2)**.**

### Early treatment by sex

**Fig. 3:**
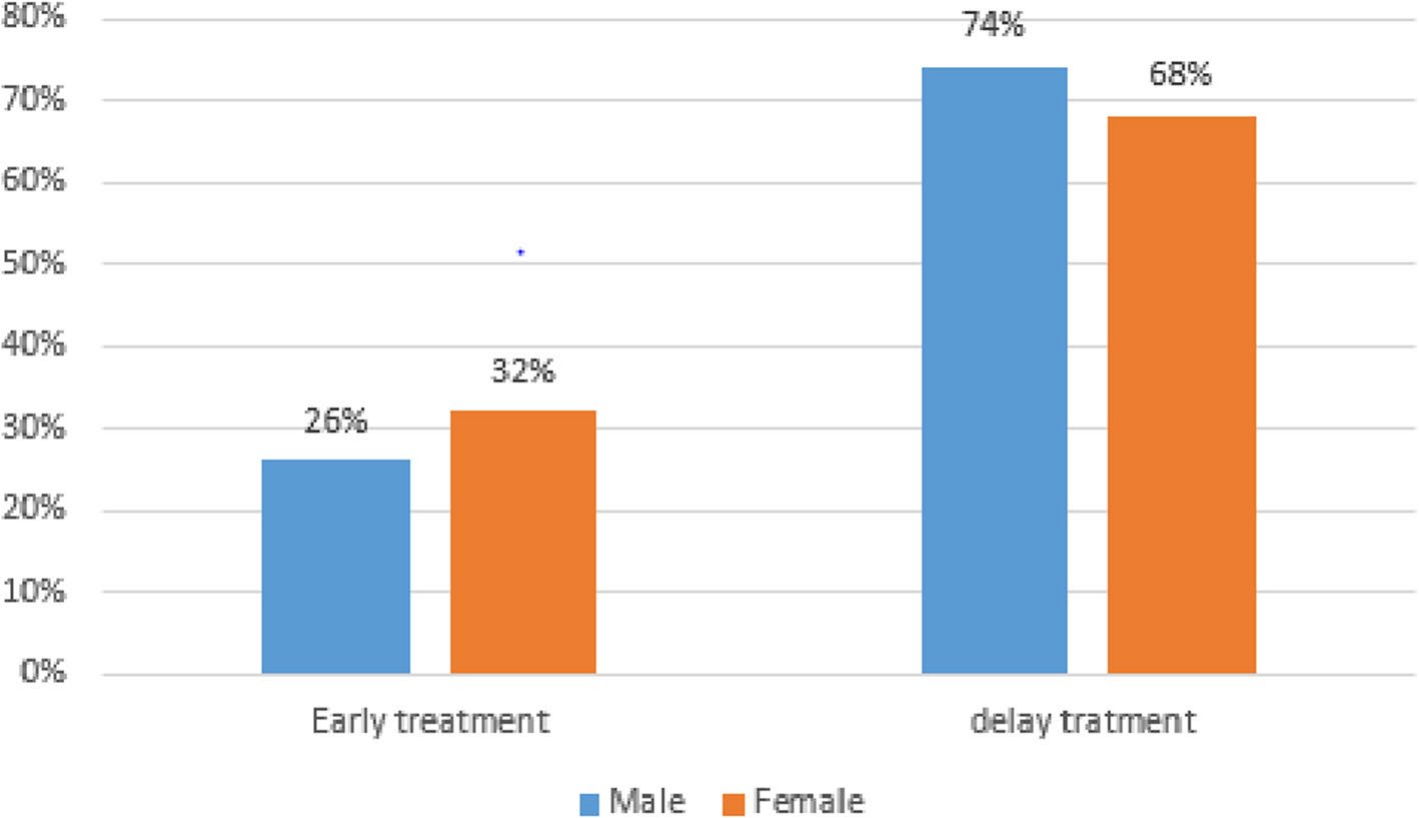
Early treatment for STI by sex among patients attending youth friendly service in Bahir Dar city health centers, northwest, Ethiopia, 2023

About 32% of the female participants were early treat STI (Fig. 3).

### Reason for delayed treatment for STI

**Fig. 4:**
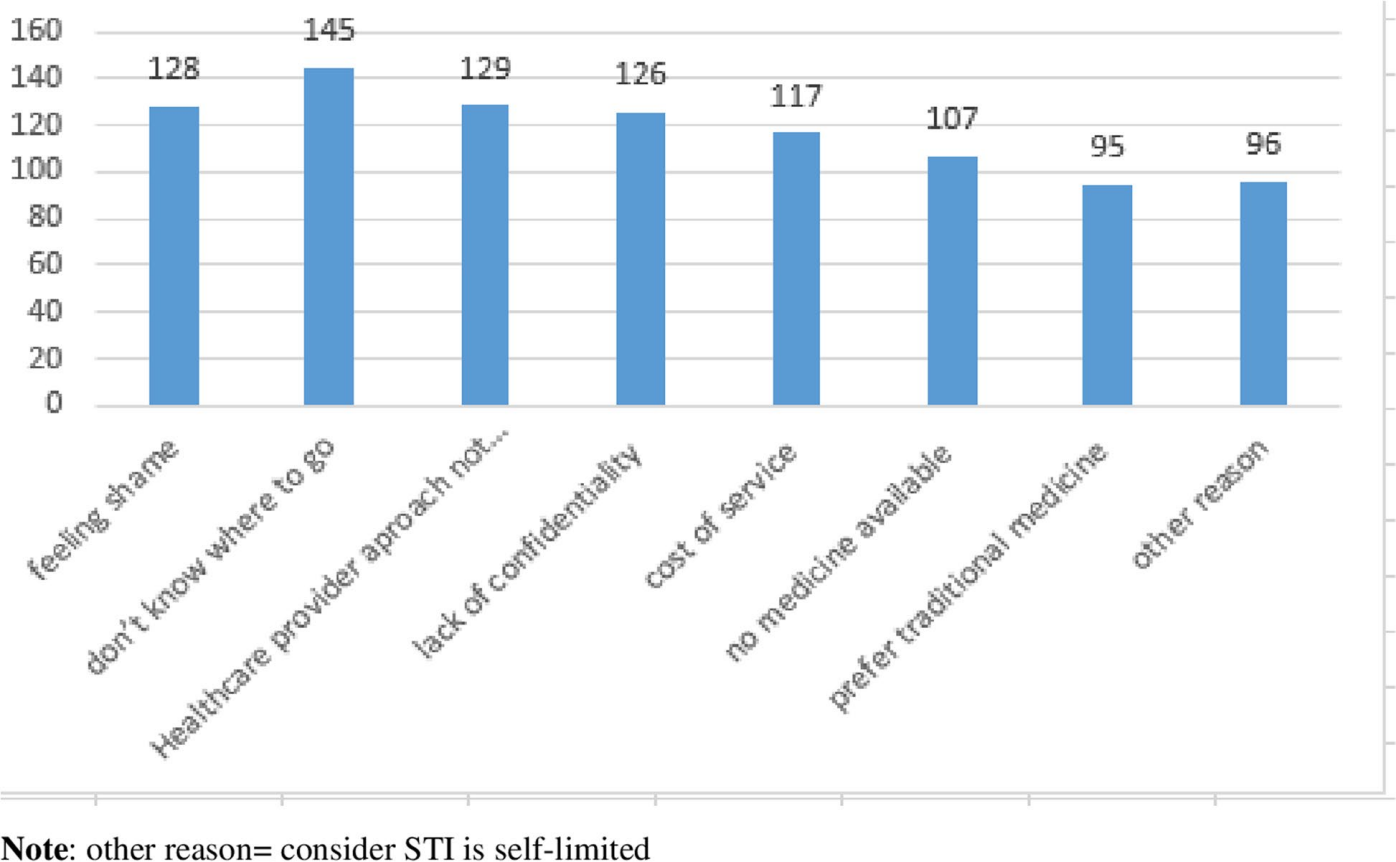
Reason for not treat early for STI among youths in Bahir Dar city health centers, Northwest Ethiopia, 2023

Among the participants who were not treated early for STI, about 145 of them were because they did not know where to go (Fig. 4).

### Factors associated with early treatment for STI

Two subsequent analyses were conducted. The first analysis was a simple binary logistic regression analysis. Of the total variables, knowledge about STIs, educational status, perception of the severity of STIs, fear of stigma, knowing HIV status, perceiving the curability of STIs, attending STI-related messages and residence were the candidate variables for multivariable binary logistic regression at a *p*-value of < 0.25. In the multivariable binary logistic regression analysis, variables such as knowledge about STI, test/ knowing HIV status, perception of the severity of STI and fear of stigma were the statistically significant variables for early treatment of STI at a *p*-value of < 0.05 and 95% CL. The model was fitted with a Hosmer and Lemishow test with a *p*-value of 0.123.

Thus, the odds of early treatment for STI among participants who had good knowledge about STI was 1.98 (AOR=1.98, 95CI%; 1.13-3.47) times more likely than their counterparts. Participants who know about their HIV status were 1.95 (AOR=1.95, 95%CI; 1.13-3.36) times more likely to treat early for STI than they did not know their HIV status. The odds of early treatment for STI among participants who perceive STI as a very serious disease were 11.2 (AOR=11.2, 95%CI; 6.15-20.45) times more likely than those who perceive STI is not a serious disease. Furthermore, participants who did not fear the stigma of being exposed to STI was 2.3 (AOR=2.3, 95%CI; 1.32-3.96) times more likely to treat early for STI than their counterparts (Table 4)**.**

**Table 4:** Bivariable and multivariable analysis of early treatment for STI among youths in Bahir Dar city health centers northwest, Ethiopia, 2023

Exposure		Treatment for STI		COR	*p*-value	AOR	*p*-value
		Yes	No				
Knowledge about STI	**Good**	78	129	3.1(1.9, 5)	<0.001	**1.98(1.1,3.5)**	0.017
	Poor	30	154	1		1	
Fear of stigma	**No**	64	91	3.1(1.9,4.9)	<0.001	**2.3(1.32,3.96)**	0.003
	Yes	44	192	1		1	
Perceive severity of STI					<0.001 (GPV)		<0.001 (GPV)
	Very serious	70	38	14.6(8.2,25.9)	<0.001	**11.2(6.2,20.4)**	<0.001
	Neutral	13	47	2.2 (1.04, 4.6)	0.038	1.97(0.9, 4.3)	0.08
	Not serious	25	198	1		1	
Know HIV status	**Yes**	66	103	2.7 (1.74, 4.34)	<0.001	**1.95(1.1,3.4)**	0.017
	No	42	180	1		1	
STI is curable	Yes	68	118	2.4 (1.5, 3.8)	<0.001	0.8(0.5,1.5)	0.551
	No	64	91	1		1	
Attend STI message	Yes	48	87	1.8 (1.1, 2.8)	0.011	1.7(0.95, 2.9)	0.073
	No	60	196	1		1	
Residence	Urban	82	160	2.4 (1.5, 4)	0.001	0.6(0.34, 1.1)	0.121
	Rural	26	123	1		1	
Educational status					<0.001(GPV)		0.31 (GPV)
	College and above	41	43	4.4 (2.4, 8.3)	<0.001	2.1(0.9, 4.6)	0.062
	Secondary	26	79	1.5 (0.8, 2.9)	0.195	1.6 (0.8, 3.6)	0.21
	Primary	19	59	1.49 (0.75, 3)	0.25	1.6 (0.7, 3.7)	0.286
	No education	22	102	1		1	

Boldly written= significantly associated

*GPV* Global *p*-value

## Discussion

In the current study, an attempt has been made to assess the proportion and its associated factors of early treatment seeking for STI among youths in Bahir Dar city. Thus, the proportion of early treatment seeking for STIs among youths who attend treatment in Bahir Dar city was 27.6% (95% CI; 23-32). Factors like perception towards STIs, knowledge about STIs, knowing their HIV status and fear of stigma were statistically significant factors for early treatment of STIs. The proportion of early treatment seeking for STI in this study was lower than a study conducted in Benishangul 40.1% [14], in South Africa 76.9% [27], in Gahanna 36% [20], in Luwero of Uganda 42% [28], another study conducted in the same country Gahanna 75.4% [15], in United States of America (70%) (41), in Asian country of Laos 58% [29], in Kerala of India 41.9% [30] and in Vietnam 20% [16]. As compared to other parts of Ethiopia, the possible reason for the discrepancy might be because of cultural and health seeking behavior difference across the region of Ethiopia. As compared to other Asian and African countries, the difference might be associated with the variation in socioeconomic, cultural, and health utilization behavior across the countries. Again the war /social unrest in Ethiopia may be also make a difference in the proportion of early treatment for STIs as compared to other countries.

Regarding the factors of early treatment seeking for STI, participants who had good knowledge about STIs were more likely to treat STIs as compared to those who had poor knowledge about STIs. This was supported by a study conducted in South Africa, Ghana, southwest Ethiopia, Benishangul Ethiopia, and Addis Ababa Ethiopia [11, 15, 17, 18]. This can be justified by knowledge is a power for wise decisions and having good knowledge about STIs complications, signs/symptoms, transmission, prevention and the advantage of early treatment enables and motivates the youth to seek the treatment earlier.

The findings of the current study also revealed that the early treatment seeking for STIs was affected by the perceived severity of STIs. That means participants who perceive STIs as a serious diseases had the higher odds of early treatment for STIs than those who perceive STIs as not a serious diseases. This was supported by a study conducted in Ghana, southwest Ethiopia [19, 20]. This might be associated with perceiving the severity of the disease or fear about the complications of STI can positively affect health-seeking behavior, as compared to those who consider STIs is not a serious disease or who perceive SIT as a self-limited disease [11, 31].

Additionally, participants who did not fear about the stigma of being exposed to STIs were more likely to treat early than their counterparts. This was supported by studies conducted in Pakistan, Nkomazi East, and rural Ethiopia [18, 26, 32]. This is because people who are not fearful of being infected with STIs are not socially embarrassed and inform their health problems early to health care providers without embarrassments [33].

Furthermore, testing/knowing the status of HIV was also affects the early treatment of STIs. This means participants who test/know their HIV status had higher odds of early treatment for STI than did not test/know their HIV status. This was supported by studies conducted in India, an African cohort study, Ethiopia and Gambela [11, 34–36]. This might be because people who know their HIV status or test for HIV have an opportunity to learn or inform about STIs from their healthcare provider. The limitation of this study was since it was a Crossectional data it shares the limitations of a cross-sessional study design. This study also not assess any difference in treatment seeking behavior of STIs between government and private health institutions. We recommended to conduct a further research on a comparative study of treatment seeking behavior of STIs in government and private health institutions.

## Conclusion

The proportion of early treatment for STI in Bahir Dar city was low. Knowledge about STIs, testing/ knowing HIV status, perception of the severity about sexually transmitted infection, and fear of stigma about STI were the statistically significant factors for early treatment of STI. So the government better give attention to health education and other health promotion activities to increase the knowledge of youths about STI and to change their perception of STI.

## Data Availability

No datasets were generated or analysed during the current study.

## Abbreviations

AOR: Adjusted Odds Ratio
CI: Confidence Interval
CL: Confidence Level
COVID-19: Corona Virus Disease
HIV: Human Immune Virus
OR: Odds Ratio
STI: Sexually Transmitted Infections
SPSS: Statistical Package for Statistical Science
WHO: World Health Organization
